# Characterization of A11 Neurons Projecting to the Spinal Cord of Mice

**DOI:** 10.1371/journal.pone.0109636

**Published:** 2014-10-24

**Authors:** Kathrin Koblinger, Tamás Füzesi, Jillian Ejdrygiewicz, Aleksandra Krajacic, Jaideep S. Bains, Patrick J. Whelan

**Affiliations:** 1 Hotchkiss Brain Institute, University of Calgary, Calgary, Alberta, Canada; 2 Department of Physiology and Pharmacology, University of Calgary, Calgary, Alberta, Canada; 3 Department of Comparative Biology and Experimental Medicine, University of Calgary, Calgary, Canada; Prince Henry's Institute, Australia

## Abstract

The hypothalamic A11 region has been identified in several species including rats, mice, cats, monkeys, zebrafish, and humans as the primary source of descending dopamine (DA) to the spinal cord. It has been implicated in the control of pain, modulation of the spinal locomotor network, restless leg syndrome, and cataplexy, yet the A11 cell group remains an understudied dopaminergic (DAergic) nucleus within the brain. It is unclear whether A11 neurons in the mouse contain the full complement of enzymes consistent with traditional DA neuronal phenotypes. Given the abundance of mouse genetic models and tools available to interrogate specific neural circuits and behavior, it is critical first to fully understand the phenotype of A11 cells. We provide evidence that, in addition to tyrosine hydroxylase (TH) that synthesizes L-DOPA, neurons within the A11 region of the mouse contain aromatic L-amino acid decarboxylase (AADC), the enzyme that converts L-DOPA to dopamine. Furthermore, we show that the A11 neurons contain vesicular monoamine transporter 2 (VMAT2), which is necessary for packaging DA into vesicles. On the contrary, A11 neurons in the mouse lack the dopamine transporter (DAT). In conclusion, our data suggest that A11 neurons are DAergic. The lack of DAT, and therefore the lack of a DA reuptake mechanism, points to a longer time of action compared to typical DA neurons.

## Introduction

Dopaminergic (DAergic) innervation of the spinal cord is thought to originate primarily from a small nucleus of tyrosine hydroxylase (TH) expressing cells in the diencephalon, also known as the A11 region [1]. Across a variety of vertebrate species, including non-human primates, zebrafish, rats, rabbits, and mice, TH-synthesizing neurons in the A11 send long axonal processes to the spinal cord [1]–[7]. Projections from the A11 region may contribute to locomotion [5]–[8], pain control [8], [9], migraines [10], and restless leg syndrome [11], yet, this cell population remains understudied in relation to other DAergic areas of the brain. In addition, these neurons were described to send projections to a wide variety of brain regions, including the dorsal raphe, thalamus, hypothalamus, and cortex [12], [13], placing the A11 in an ideal position to potentially provide an alternative motor innervation to locomotor circuits compared to the well-described DAergic substantia nigra pars compacta-striatal network.

Over the last decade, there has been a greater appreciation that the classification of DAergic nuclei is more diverse than first thought [14]–[17]. DAergic neurons contain TH to convert L-tyrosine into L-DOPA and aromatic L-amino acid decarboxylase (AADC) that synthesizes dopamine (DA) from L-DOPA. DA neurons also express vesicular monoamine transporter 2 (VMAT2) to package the DA into vesicles as well as dopamine reuptake transporters (DAT) to clear DA from the synaptic cleft. Interestingly, there are examples of monoenzymatic neurons that contain either TH or AADC [4], [15], [18]. There are also examples of neurons that are bienzymatic but lack VMAT2, DAT, or both [16], [19]. These types of cells are dispersed throughout the brain including the hypothalamus, and identification of their phenotype is critical if we are to unravel downstream signaling mechanisms [16]. The A11 region remains incompletely characterized in most species making it difficult to determine whether or not the ascribed functional effects are due to DA. For example, A11 neurons in the non-human primate are positive for TH but lack DAT and AADC [4]. Furthermore, there seems to be species-differences in regards to D_2_ autoreceptors in A11, which is significant since they contribute to the activity of DA neurons and synthesis of DA itself [20], [21]. Therefore, it remains uncertain in the mouse whether A11 neurons are DAergic or not.

Given the wide use of DA mouse models, it is critical to examine the phenotype of A11 neurons in this species. The main aims of this study are to characterize the phenotype of mouse A11 neurons, their projections to the spinal cord, and whether they contain the full or a partial set of enzymes required for DA synthesis and release. Here we provide evidence that A11 neurons are AADC, TH and VMAT2 positive but are negative for DAT expression. Portions of this study have been published in abstract form [22].

## Materials and Methods

### Ethics statement

This work was completed in accordance with the recommendations in the Canadian Council for Animal Care. The protocol was approved by the Health Sciences Animal Care Committee of the University of Calgary (Protocol Number: M11028). Every effort was made to minimize animal suffering, and all surgeries were completed using isoflurane gas anesthesia. All surviving animals were given a buprenorphine injection post-surgery.

### Animals

Tyrosine Hydroxylase (TH)-Cre transgenic mice (Source: Jackson Laboratory, B6.Cg-Tg (Th-Cre)1Tmd/J, Stock Number:008601; n = 5) and wildtype C57/Bl6 mice (Source: Jackson Laboratory, C57BL/6J, Stock Number:000664; n = 10) were used. All mice were between the ages of 6–12 weeks old throughout the study. Mice were housed under controlled lighting conditions (12∶12 light∶dark cycle) with food and water available *ad libitum*. Only male mice were used in this study due to reported sex differences in DA concentrations in the spinal cord of mice [21], [23] and to exclude possible variability in DA expression due to estrus cycles.

### FluoroGold injections

Mice were anesthetized with isoflurane (1.5%) and secured in a stereotaxic frame (Stoelting Co., IL, USA) positioned dorsally upwards. A laminectomy was performed between lumbar vertebral segments (L4 to L5) followed by a careful removal of the dura mater to expose the dorsal surface of the spinal cord. The fluorescent tracer FluoroGold (FG; 2% w/v in 100 µL Saline; Fluorochrom) was pressure injected (Nanoject II Nanoliter Injector, Drummond Scientific, PA, USA) bilaterally (70 nl per injection, 3 bilateral injections, total of 6 injections) into the spinal cord through a glass capillary pulled to a fine tip (3.5″ glass capillaries, Drummond Scientific, PA, USA; Puller Narishige, diameter 15–20 µm). The muscles overlying the vertebra and skin were then sutured and the mice were given buprenorphine (0.1 mg per kg) for post-surgery analgesia. Animals were sacrificed 14 to 16 days after surgery to conduct retrograde FG transport analysis throughout the spinal cord.

### Yellow Fluorescent Protein injections

Animals were anesthetized following the same protocol for FG injections with minor differences. The heads of the mice were positioned in a stereotaxic frame with Bregma and Lambda landmarks positioned in the horizontal plane of the apparatus. Through a small burr hole drilled vertically through the skull (Dremel 300, drill bit 105, Robert Bosch Tool Corporation, IL), a 3.5″ glass capillary tube (Drummond Scientific, PA, USA; Puller Narishige) with a tapered tip (diameter 15–20 µm) was lowered into the brains at stereotaxic coordinates corresponding to the A11 nucleus (anteroposterior (AP), −2.3 mm; mediolateral (ML), 0.2 mm; dorsoventral (DV), 3.0 mm [24]). Five TH-Cre mice received pressure injections bilaterally of the recombinant AAV vector carrying Yellow Fluorsecent Protein (YFP) (AAV2/1.EF1aDIO.EYFP.WPRE.hGH Addgene plasmid 27056, Penn State Vector Core) with a total volume of 420 nl (2.16×10^12^ GC ml^−1^). YFP injections followed 3 days after the FG spinal cord injections at an age of 10 to 11 weeks (n = 4).

### Immunohistochemistry

To prepare fixed brain tissue, mice were anesthetized with isoflurane and perfused transcardially with 0.1 M phosphate-buffer saline (PBS, pH 7.4, 20°C) followed by 4% paraformaldehyde (PFA) in phosphate buffer (PBS, 4°C). Brains and spinal cords were removed, submerged in PFA at 4°C for 24 hours then cryoprotected in 30% sucrose (0.1 M PBS) for 24 to 48 hours at 4°C. 30 µm coronal brain sections, as well as sagittal and transverse spinal cord sections, were frozen in clear frozen section compound (OCT - VWR International, Radnor, PA, USA) and sectioned using a cryostat (Leica CM1850 UV, Leica Biosystems, Ontario, Canada). The sliced brain sections were collected in a staggered fashion and either placed into four consecutive wells to free-float in PBS or mounted staggered onto six Superfrost Plus slides (VWR International). Spinal cord slices were mounted across 6 slides also in a staggered fashion. All slides were warmed on a hot plate (35°C for 20 min) before washing. The brain and spinal cord sections (either mounted or free-floating) were washed in 0.1 M PBS before and between incubations, followed by one 20 min wash in PBS with 0.5% Triton-×100, and lastly by one 30 min period in blocking solution (5% goat and 5% donkey serum in PBS). The blocking solution was also used in subsequent antibody incubations. Once the immuno incubations were complete, free-floating coronal brain sections were then mounted onto Superfrost Plus slides. All slides were then lightly coated with Fluoromount/Plus medium (Diagnostic Biosystems, Pleasanton, CA, USA) and coverslipped. All primary and secondary antibodies, along with their conjugates, are presented in Table 1 and 2, respectively. To test for nonspecific binding of the secondary antibody, we excluded the primary antibodies completely throughout the study. No significant background fluorescence intensity was observed in the brain sections with secondary antibodies alone.

**Table 1:** Primary Antibodies.

Antigen	Lab Code	Commercial Source	Dilution Incubation time	Donor Species
TH	AB112	Abcam Inc., Toronto, ON, Canada	1∶1000 - overnight at room temperature	Rabbit
TH	AB 1542	EMD Millipore, Billerica, MA, USA	1∶500 - overnight at room temperature	Sheep
AADC	NBP1-56918	Novus Biologicals, Littleton, CO, USA	1∶500 - 60 hours at 4 degrees C	Rabbit
VMAT2	H-V008	Phoenix Pharmaceuticals, Burlingame, CA, USA	1∶500 - 60 hours at 4 degrees C	Rabbit
DAT	MAB369	EMD Millipore, Billerica, MA, USA	1∶500 - 60 hours at 4 degrees C	Rat
FG	AB153	EMD Millipore, Billerica, MA, USA	1∶1000 - overnight at room temperature	Rabbit
GFP	GFP-1020	Aves Laboratories, Tigard, OR, USA	1∶500 - overnight at room temperature	Chicken

**Table 2:** Secondary Antibodies.

Secondary Antibody	Dilution	Commercial Source
Biotinylated donkey anti-rabbit	1∶500	Jackson Immuno Research, West Grove, PA, USA
Cy3-conjugated strepavidin	1∶500	Jackson Immuno Research, West Grove, PA, USA
Alexa-564-conjugated donkey anti-rabbit	1∶500	Molecular Probes, Burlington, ON, Canada
Alexa-488-conjugated donkey anti-sheep	1∶500	Molecular Probes, Burlington, ON, Canada
Alexa-564-conjugated donkey anti-rat	1∶500	Molecular Probes, Burlington, ON, Canada
Alexa-488-conjugated donkey anti-rabbit	1∶500	Molecular Probes, Burlington, ON, Canada
Alexa-564-conjugated goat anti-rabbit	1∶1000	Molecular Probes, Burlington, ON, Canada
Alexa-488-conjugated goat anti-chicken	1∶500	Molecular Probes, Burlington, ON, Canada

Confocal fluorescent images were collected using a Nikon Eclipse C1si Spectral Confocal microscope with a motorized stage (Nikon Canada Inc., Ontario, Canada). The objectives used were 10× Plan Apo DIC (NA 0.45), 20× Plan Apo DIC (NA 0.75), Nikon Plan Fluor 40× oil immersion DIC (NA 1.30), and 60× Plan Apo water immersion DIC (NA 1.20). The lasers used were centered on 488 nm (with a 515/30 emission filter) and 561 nm (with a 590/50 emission filter) wavelengths. The 10× images were taken with z-step 1 µm, 20× with z-step 0.5 µm, 40× with z-step 0.3 µm, and 60× with z-step 0.15 µm, all using the imaging software EZ-C1 for Nikon Confocal, Silver Version 3.91 (Nikon Canada Inc., Ontario, Canada). Stacked images were acquired by averaging 4 frames with a resolution of 1024×1024 or 512×512. Off-line image processing included maximal intensity projections conducted using NIS-Elements Advanced Research Version 4.10 as well as adjustments of brightness and contrast in Photoshop. For images illustrating DAT the despeckle function was used to decrease noise and improve contrast. The same adjustments and settings were employed for all images within a given series for image consistency and comparison.

We assessed the entire rostrocaudal extent of the A11 region. In randomly selected sections, the diameter of TH positive cells in 20× confocal images were measured by a person blinded to the study (rostral section 23 cells, middle section 27 cells, caudal section 43 cells). TH and AADC co-localizing cells were counted using 20× confocal images. Cell counting was accomplished using ImageJ (NIH Image, Bethesda, MD, USA).

### Data analysis

Data are reported as means ± SEM. A one-way ANOVA was used to detect differences among groups (GraphPad Prism 6.0). P<0.05 was considered statistically significant.

## Results

The overall aim of the current work was to examine whether A11 neurons projecting to the spinal cord contained AADC, TH, VMAT2 and DAT consistent with the synthesis and reuptake of DA.

### Characterization of TH expressing neurons within the A11

**Figure 1:**
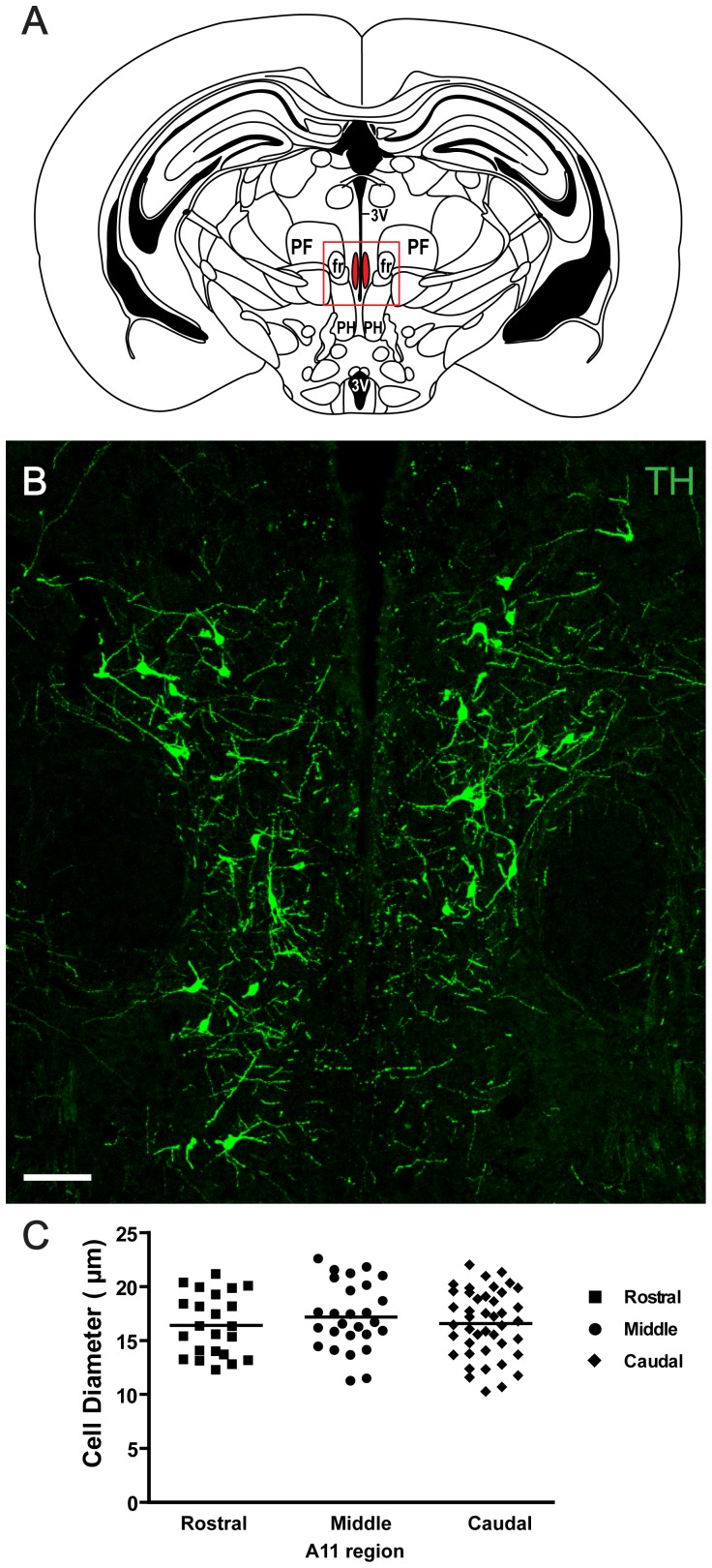
Characterization of tyrosine hydroxylase (TH) positive cells in A11. A: Diagram showing the middle region of the A11 area in the mouse. The red frame represents the area where representative micrographs were taken. The diagram was adapted from [24] with permission. B: TH immunohistochemistry (green) of the middle region of A11. Scale bar 100 µm. 3 V = third ventricle, PF = parafascicular thalamic nucleus, fr = fasciculus retroflexus, PH = posterior hypothalamic nucleus, mt = mammillothalamic tract. C: Diameter of TH positive cells in three regions - rostral (AP −2.0 mm), middle (AP −2.3 mm) and caudal region (AP −2.5 mm). The mean cell diameter was 16.7±0.3 µm and the cell diameters between sections were similar.

A11 neurons were first characterized in terms of size and location (Fig. 1A). TH-IR A11 cells were found in the diencephalon, extending dorsally along the periventricular grey matter of the caudal thalamus reaching medially to the mamillothalamic tract. In accordance with other reports, A11 cells were found to be relatively large (>16 µm) multipolar cells compared to non-TH-IR cells in the vicinity (Fig. 1B).

The A11 was divided into three regions in this study - rostral (anteroposterior (AP), −2.0 mm), middle (AP, −2.3 mm) and caudal region (AP, −2.5 mm) [24]. The A11 region began approximately 2.0 mm caudal to Bregma where the cells cluster bilaterally near the midline, dorsal to the third ventricle and medial to the mamillothalamic tracts (Fig. 1). Moving rostrally to the middle region of A11, neurons were located more dorsally, surrounding the third ventricle, and medially to the fasciculus retroflexus (Fig. 1A). Cells in the caudal region were located ventral to posterior commissure (PC) on both sides of the third ventricle.

The mean cell diameter was 16.7±0.3 µm (rostral 16.4±0.6 µm, middle 17.2±0.6 µm, caudal 16.6±0.47 µm) and the cell diameters between sections were very similar (F(2,90) = 0.49, p = 0.62) (Fig. 1C).

### TH-IR A11 cells project to the spinal cord

**Figure 2:**
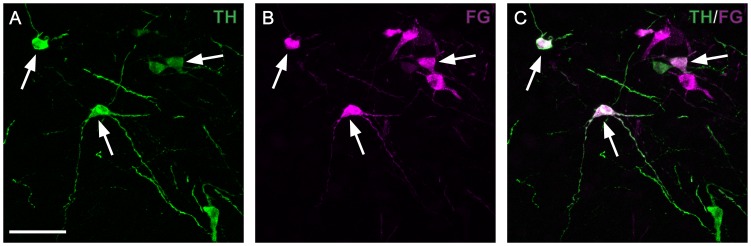
A11 TH positive neurons project to the spinal cord. Example of retrograde labelling in the A11 caudal area following FluoroGold (FG) injections into the lumbar spinal cord between lumbar vertebral segments L4 and L5. Representative double-fluorescent immunostaining of tyrosine hydroxylase (TH; green) and FluoroGold (FG; magenta). The white arrows point to double-labeled cells. Most of the TH positive cells also contain FG (arrows), one cell shows labelling for TH but not FG. Several cells that contain FG but not TH are also seen (not co-localizing). Scale bar: 50 µm.

To determine whether TH cells labeled in the putative A11 area projected to the spinal cord, the FG retrograde tracer was nanoinjected into the spinal cord between lumbar vertebral segments L4 and L5 (Fig. 2). This area was chosen since it contains a relatively large proportion of neuronal targets. After allowing 14 to 16 days for retrograde transport, the retrogradely-labeled neurons dorsal to the hypothalamus showed typical cytoplasmatic granular staining observed using FG (Fig. 2B). Double-labeled TH-IR neurons were located within the A11 cell group (Fig. 2C). In addition, the double-labeled neurons were located bilaterally throughout the entire rostrocaudal extent of the A11.

**Figure 3:**
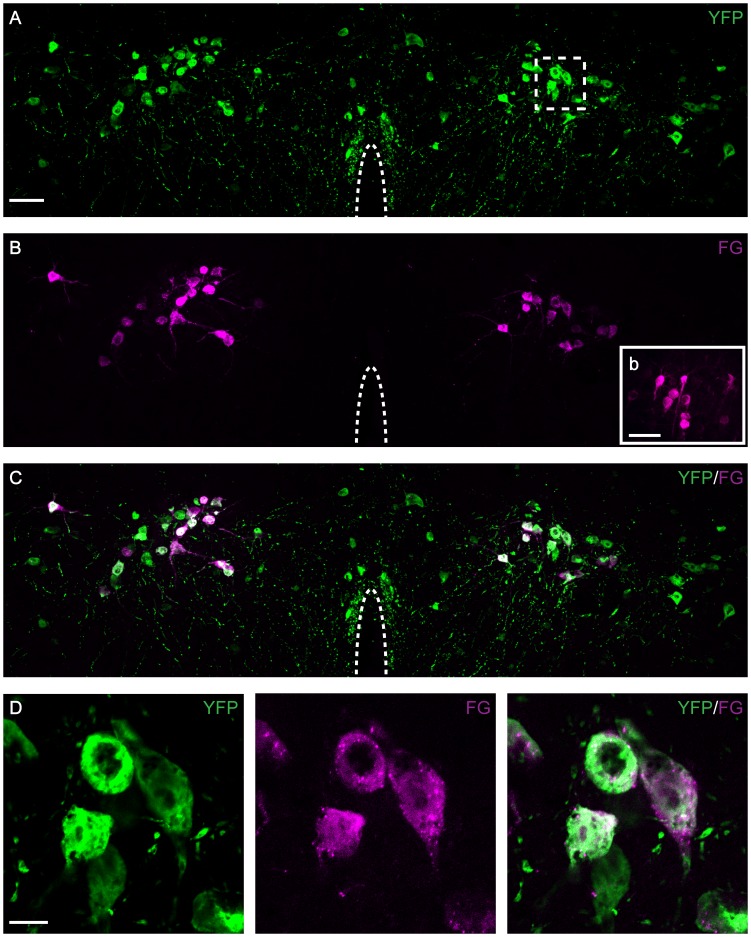
YFP expression in A11 neurons following Cre-dependent viral expression and retrograde tracing with FG from the spinal cord. Rostral region section of A11 from a TH-Cre mouse transduced with a Cre-dependent YFP AAV (green) and immunohistochemistry against FG (magenta) (A–D). Boxed inset in (B (b)) shows FG labeled cells in the motor cortex. Only cells in A11 were co-labeled with YFP and FG (C, D). Scale bars: 50 µm. D: Higher magnification of boxed area in A. Scale bar: 20 µm.

To further investigate the connectivity of A11 to the spinal cord, adeno associated virus (AAV) containing an YFP double-floxed construct was injected into the A11 region of transgenic TH-Cre mice. FG was also injected into the spinal cord within the lumbar 4 and 5 segments. To maximize the detection of YFP in fibers, immunohistochemistry was performed - using an anti-GFP antibody – which can detect YFP. Strong YFP expression was observed in the perikarya of TH-Cre neurons in the area of A11 (Fig. 3A). Depending on the location of the virus injection, expression was observed in either the rostral and middle or middle to caudal regions of the A11. In the A11 region, retrogradely-labeled FG containing neurons co-localized with YFP (Fig. 3C, D). This approach provided additional evidence for the TH phenotype of the A11 and indicated that TH neurons located in the A11 project to the spinal cord. As a control, cells were observed in the motor cortex where, as expected, FG-labeled neurons lacked YFP expression (Fig. 3Bb (inset)).

**Figure 4:**
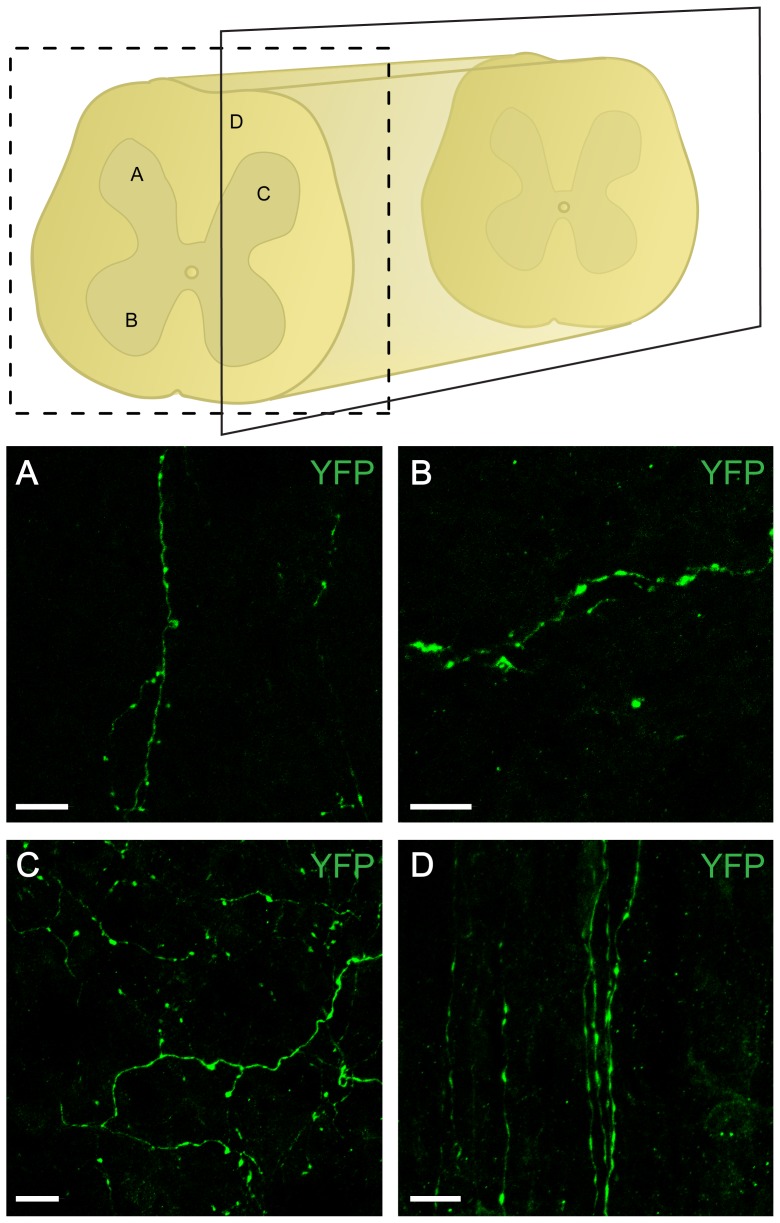
YFP positive fibers in the lumbar spinal cord originating from A11. Schematic of fiber localization in the lumbar spinal cord. Representative micrographs of YFP-labeled fibers in transverse (A, B) and parasagittal (C, D) lumbar spinal cord sections. Fibers were found in the dorsal (A) and ventral horn (B) as well as in the grey (C) and the white matter (D). Scale bar: 10 µm.

In the spinal cord, YFP-IR fibers were observed at lumber segments L1to L3 (within our targeted area) in the white matter (Fig. 4D) and were also dispersed throughout the grey matter (Fig. 4A, B, C). YFP-IR collaterals also projected into the grey matter from the white matter (data not shown). Consistent with previous reports, longitudinal YFP-IR fibers running rostrocaudally in the white matter were also apparent (Fig. 4D). Within the grey matter, YFP^+^ fibers were found equally dispersed in most Rexed's laminae. A subpopulation of fibers in the L1 to L3 segments was positive for both YFP and FG.

### A11 TH-IR neurons contain AADC

**Figure 5:**
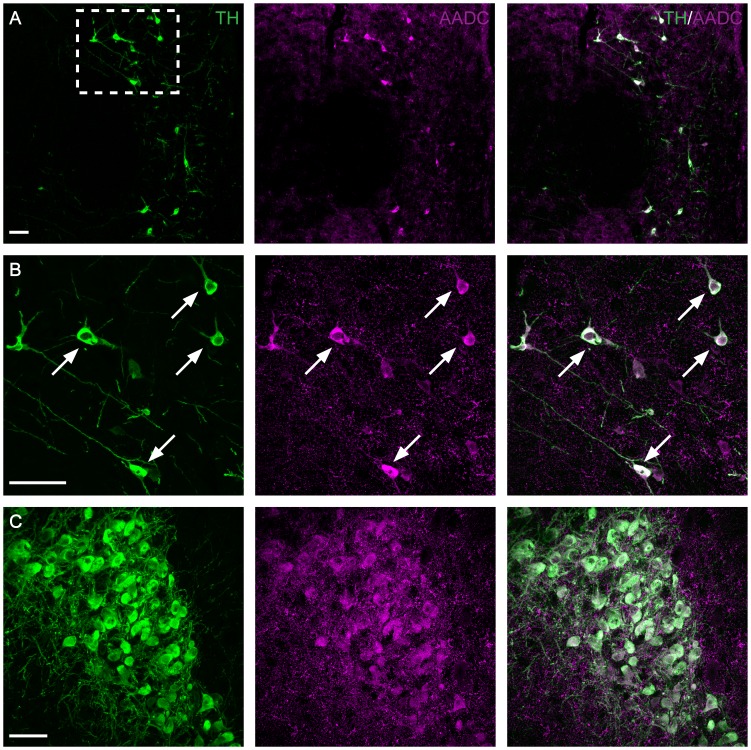
A11 TH positive neurons are also expressing aromatic amino acid decarboxylase (AADC). Immunohistochemistry targeted against TH (green) and AADC (magenta) shown in a representative middle region section of A11 (A, B) and the locus coeruleus (C). Note the co-localization between TH and AADC positive neurons (arrows) in A11 (B). Locus coeruleus served as a positive control (C). Scale bar 50 µm. A and B are derived from data shown in Figure 1. B: Higher magnification of boxed area in A.

To further characterize the phenotype of A11 neurons, immunohistochemistry to detect TH and AADC was performed. The locus coeruleus was also processed as a positive control since it is known to contain AADC synthesizing noradrenergic neurons [25], [26]. In the A11, TH-IR neurons were mostly labeled for AADC, suggesting that these neurons contain the enzymatic machinery necessary to convert L-DOPA to DA (Fig. 5A, B). Of 338 counted cells (100 cells in the rostral region, 108 in the middle and 130 in the caudal region) that were positive for TH, 98.9±1.08% (n = 4 animals) were immunopositive for AADC. As expected, AADC expression in the locus coeruleus was intense and was co-localized with TH (Fig. 5C).

### TH-IR A11 cells contain the vesicular transporter VMAT2

**Figure 6:**
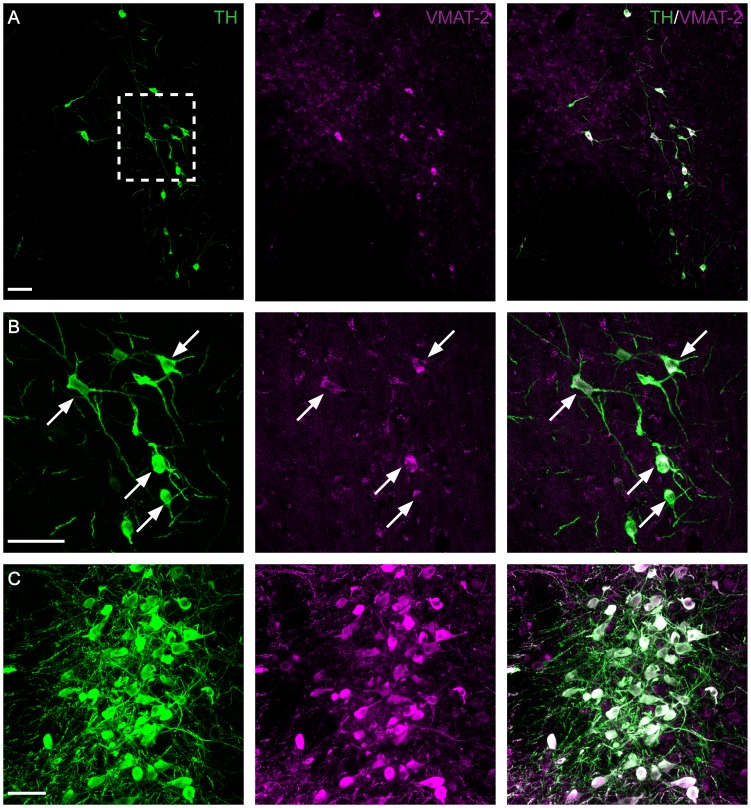
Vesicular monoamine transporter (VMAT-2) expression in A11 and locus coeruleus. Immunohistochemistry targeted against TH (green) and VMAT-2 (magenta) in a representative micrograph of a middle region section of A11 (A, B). Note the co-localization between TH and VMAT-2 indicated by the arrows (B). Locus coeruleus served as a positive control (C). B: Higher magnification of boxed area in A. Scale bar: 50 µm.

To test whether A11 TH-IR neurons also contained VMAT2, an antibody against VMAT2 was utilized (Fig. 6). VMAT2 labeling was granulated and more intense around the cell bodies (Fig. 6B, centre panel). A11 neurons were found to co-label almost exclusively for TH and VMAT2 (Fig. 6A, B) even though VMAT2 labeling appeared less intense than in the locus coeruleus that served as a control (Fig. 6C).

### TH-IR A11 cells do not contain DAT

**Figure 7:**
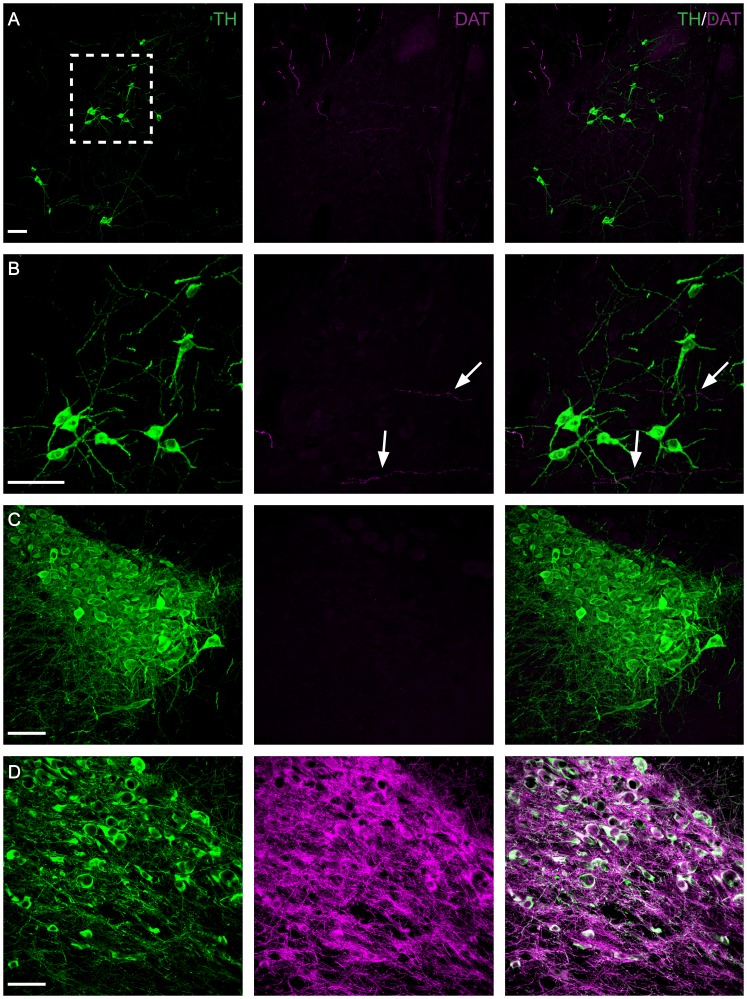
Absence of dopamine transporter (DAT) expression in A11. Immunohistochemistry targeted against TH (green) and DAT (magenta) (A–D). Representative micrographs showing the absence of DAT labeling in the middle region of A11 (A, B). Note the DAT positive but TH negative fibers in A11 (B) (arrow). (C) As expected DAT is absent in the locus coeruleus (LC) which served as a negative control. Representative micrograph of TH and DAT labeled neurons and fibers in the ventral tegmental area (VTA) (D). Note the absence of DAT expression in the A11 and the LC in contrast with the intense expression in the VTA (positive control). B: Higher magnification of boxed area in A. Scale bar: 50 µm.

**Figure 8:**
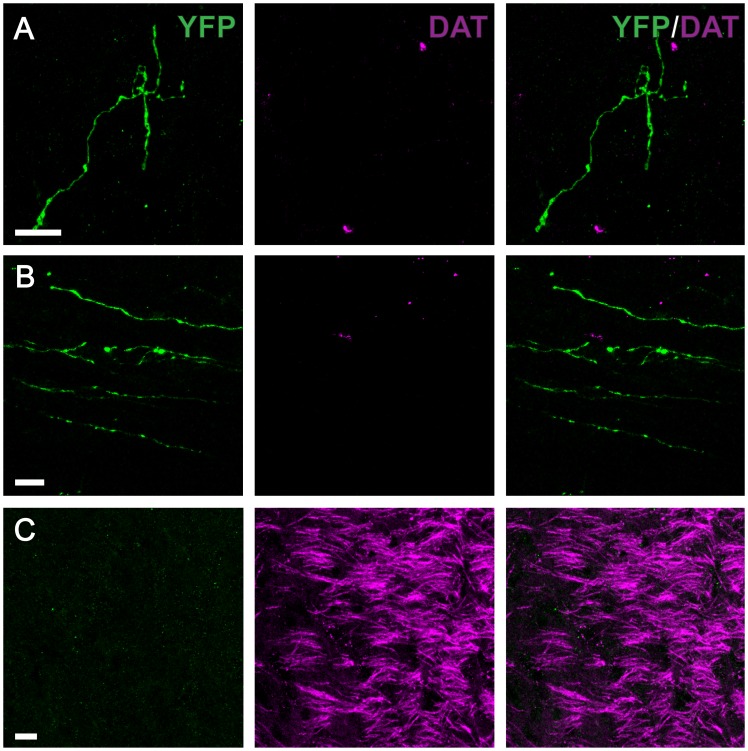
YFP positive fibers in the lumbar spinal cord originating from A11 lack dopamine transporter (DAT). Immunohistochemistry targeted against YFP (green) and DAT (magenta) (A–C). Representative micrographs showing YFP-labeled fibers in parasagittal lumbar spinal cord sections in the grey (A) and white (B) matter. (C) Positive control showing intense expression of DAT in the substantia nigra compacta. Scale bar: 10 µm.

The presence of DAT is often used to define DAergic neurons [16], [27]. To determine whether TH-IR A11 neurons are DAT-IR, immunohistochemistry for TH and DAT on diencephalic brain slices was performed. It was observed that A11 TH-IR cell bodies were uniformly lacking DAT− immunoreactivity (Fig. 7A, B). A few single fibers were found to be DAT-IR but these were not TH^+^ (see white arrows Fig. 7B). As an additional control the locus coeruleus was also examined and found to be, as expected, DAT^−^ (Fig. 7C). Neurons and fibers in the ventral tegmental area (VTA) were strongly positive for DAT and co-localized with TH (Fig. 7D). Another possibility is that DAT was localized in the terminals of A11 neurons. The presence of DAT was stained for in mice injected with AAV containing floxed YFP. This revealed no evidence of YFP^+^ projecting fibers containing DAT in either the gray or white matter of the spinal cord (Fig. 8A, B). However, positive controls were performed in the substantia nigra that were found to be strongly DAT^+^ (Fig. 8C).

## Discussion

The work presented here shows, for the first time, that the A11 cell group contains sufficient enzymatic machinery to generate dopamine in the mouse. In particular, we demonstrate that TH-positive neurons contain AADC, the enzyme necessary to produce DA, and VMAT2 that is essential for the packaging of DA into vesicles for synaptic release. This agrees with evidence demonstrating that stimulation of the A11 produces sensorimotor effects that are dependent on DA receptor activation. In summary these data provide evidence for a class of neurons in the A11 that partly exhibit a DA phenotype.

Notably, we did not find evidence for the presence of DAT in TH^+^ regions of the A11 or in YFP^+^ projections in the spinal cord, which confirms previous work done in the non-human primate [4], human [28], and rat [29]. Data from the Allen Mouse Brain Atlas also shows no evidence for DAT in the vicinity of A11 (http://mouse.brain-map.org/). However, we did see evidence for some DAT fibers in the A11 but these did not co-localize with TH. Compared to the VTA, which served as a positive control, the absence of DAT in the A11 was striking. The absence of DAT is also consistent with other work showing a lack of this enzyme particularly in hypothalamic DA cell groups [29], [30]. Furthermore, the presence of non-DAT containing TH positive neurons has been reported in several nuclei, including the periventricular nucleus (A14), preoptic area (A15), and supraoptic nucleus [29].

While DAT is associated with a characteristic DAergic phenotype, it is not necessary for the release of DA. This is clear from work utilizing DAT knockout mice. These mice are viable, and have increased DAergic tone with pathologies typical of DA overproduction [31]. As a result, it is reasonable to speculate that DA could be released from A11 projecting terminals with no reuptake. Our data are consistent with others showing that a significant proportion of A11 TH neurons project to the spinal cord, contain long processes, and show relatively sparse innervation when transverse sections of the spinal cord were examined [1], [6], [7], [32], [33].

This raises the issue of the functional effects of DA. While we do not know whether or not DA is released in a paracrine fashion within the spinal cord, it is reasonable to speculate that, similar to 5-HT and noradrenaline (NA), it would be [34]. 5-HT and NA show evidence for paracrine release mainly in the dorsal horn [35], [36]. Furthermore, work from other areas of the brain, including the dorsal raphe nucleus and the substantia nigra, show evidence for both junctional and non-junctional DAergic transmission [37]. The lack of DAT in knock-out animals is associated with a five-fold increase in extracellular DA concentrations and dramatically increases the lifetime of extracellular DA in the striatum [38]. Even with functional reuptake, volume transmission of 5-HT within both the spinal cord and dorsal raphe can reach the order of 20 µm. Coupled with a half-life of 200 ms, this suggests volume-released monoamines have considerable effects on both pre- and post-synaptic targets. One caveat is that DA reuptake is known to occur in the noradrenaline transporter (NET) rather than DAT. In the frontal cortex and hippocampus, DA reuptake appears to occur primarily by NET while it occurs via DAT in the caudate and nucleus accumbens [39], [40]. This suggests that the absence of DAT does not always indicate a lack of DA reuptake. However, in the case of DA via NET, reuptake has been reported to be an order of magnitude slower compared to traditional DAT reuptake.

Our data suggest that A11 neurons in the mouse contain all the enzymatic machinery (TH and AADC) necessary to produce DA, but also contain transporters (VMAT2) to package DA into vesicles. Even though the A11 nucleus may release DA, there are still several questions unaddressed regarding its function. In various DA neuronal systems, DA synthesis is regulated by presynaptic inhibitory D_2_ autoreceptors that limit intracellular DA concentrations. The absence of D_2_ autoreceptors suggest higher DA release probabilities [20]. Within the A11 of the rat there appears to be no evidence for any DA receptors [10], while there is no evidence for D_2_ autoreceptor regulation of dopamine release in the mouse [21]. Finally, in the non-human primate there is no evidence for AADC expression suggesting the possibility of species-differences in A11 neurotransmitter phenotype. There is also some evidence that A11 neurons may release both glutamate and DA [41]. Nevertheless only a sub-population of A11 cells in the rat contain vesicular glutamate transporter 2 (VGlut2) [41]. While we don't know the interactions within the spinal cord, interactions between DA and glutamate have been reported in the terminal fields of A10 DA neurons that also contain VGlut2 [42], [43]. A paucity of co-localization within the same varicosity for either A10 or A11 has been reported suggesting that DA and glutamate release may occur from adjacent varicosities. Clearly, future work is required to tease apart these possibilities.

From a functional viewpoint, several lines of evidence suggest that DA modulates spinal reflex and locomotor circuits, and has clinically been suggested that ‘restless leg syndrome’ is due to a reduction of spinal release of DA [11], [44]–[48]. In the neonatal rat and mouse, our work, and that of others, shows that bath application of DA restricted to the thoracolumbar spinal region modulates fictive locomotor patterns [47]–[53]. This DA excitation of mammalian spinal locomotor circuits appears to occur primarily through D_1_ receptors [48], [54]–[57]. Microdialysis measurements show release of DA and its metabolites in the ventral horn of adult rats following walking [58]. Moreover, administration of D_1_ agonists in adult mice with a complete thoracic injury elicited bouts of stepping [59]. Finally, the key role played by DA transmission in locomotion is highlighted by the fact that L-DOPA-elicited air stepping in intact neonatal rats can be blocked by intrathecally introduced DA receptor antagonists [60], [61]. The low levels of DA suggest that it may operate similar to the actions of trace amines (TA) within the spinal cord [62]. Trace amine receptors (TAR) have been found in the spinal cord, and DA can activate trace amines. TARs have been found to be expressed on multiple classes of cells in the spinal cord including D cells, motoneurons, and other interneurons within the spinal cord (Hochman, personal communication). In short, TARs are in a position to amplify the effects of limited levels of DA within the spinal cord and, in concert with a lack of reuptake on A11 projecting neurons, could help explain the disparity between anatomical and functional studies.

## References

[1] Qu S, Ondo WG, Zhang X, Xie WJ, Pan TH, et al (2006) Projections of diencephalic dopamine neurons into the spinal cord in mice. Exp Brain Res 168: 152–156 10.1007/s00221-005-0075-1 16044299

[2] Lambert AM, Bonkowsky JL, Masino MA (2012) The conserved dopaminergic diencephalospinal tract mediates vertebrate locomotor development in zebrafish larvae. J Neurosci 32: 13488–13500 10.1523/JNEUROSCI.1638-12.2012 23015438 PMC3481997

[3] Hökfelt T, Phillipson O, Goldstein M (1979) Evidence for a dopaminergic pathway in the rat descending from the A11 cell group to the spinal cord. Acta Physiologica Scandinavica 107: 393–395 10.1111/j.1748-1716.1979.tb06491.x 396764

[4] Barraud Q, Obeid I, Aubert I, Barrière G, Contamin H, et al (2010) Neuroanatomical Study of the A11 Diencephalospinal Pathway in the Non-Human Primate. PLoS ONE 5: e13306 10.1371/journal.pone.0013306 20967255 PMC2954154

[5] Blessing WW, Chalmers JP (1979) Direct projection of catecholamine (presumably dopamine)-containing neurons from hypothalamus to spinal cord. Neurosci Lett 11: 35–40. 431883 10.1016/0304-3940(79)90052-1

[6] Skagerberg G, Lindvall O (1985) Organization of diencephalic dopamine neurones projecting to the spinal cord in the rat. Brain Res 342: 340–351. 4041835 10.1016/0006-8993(85)91134-5

[7] Lindvall OL, Bjrklund ABR, Skagerberg G (1983) Dopamine-containing neurons in the spinal cord: anatomy and some functional aspects. Ann Neurol 14: 255–260 10.1002/ana.410140302 6314870

[8] Fleetwood-Walker SM, Hope PJ, Mitchell R (1988) Antinociceptive actions of descending dopaminergic tracts on cat and rat dorsal horn somatosensory neurones. J Physiol 399: 335–348. 2841456 10.1113/jphysiol.1988.sp017084 PMC1191668

[9] Pappas SS, Kennedy T, Goudreau JL, Lookingland KJ (2011) Opioid-mediated regulation of A11 diencephalospinal dopamine neurons: pharmacological evidence of activation by morphine. Neuropharmacology 61: 614–621 10.1016/j.neuropharm.2011.05.002 21605572 PMC3130120

[10] Charbit AR, Akerman S, Holland PR, Goadsby PJ (2009) Neurons of the dopaminergic/calcitonin gene-related peptide A11 cell group modulate neuronal firing in the trigeminocervical complex: an electrophysiological and immunohistochemical study. J Neurosci 29: 12532–12541 10.1523/JNEUROSCI.2887-09.2009 19812328 PMC6665099

[11] Clemens S, Rye D, Hochman S (2006) Restless legs syndrome: revisiting the dopamine hypothesis from the spinal cord perspective. Neurology 67: 125–130 10.1212/01.wnl.0000223316.53428.c9 16832090

[12] Peyron C, Luppi PH, Kitahama K, Fort P, Hermann DM, et al (1995) Origin of the dopaminergic innervation of the rat dorsal raphe nucleus. Neuroreport 6: 2527–2531. 8741755 10.1097/00001756-199512150-00019

[13] Kagan R, Kainz V, Burstein R, Noseda R (2013) Hypothalamic and basal ganglia projections to the posterior thalamus: Possible role in modulation of migraine headache and photophobia. Neuroscience 248C: 359–368 10.1016/j.neuroscience.2013.06.014 PMC3858508 23806720

[14] Dahlström A, Fuxe K (1964) Evidence for the existence of monoamine-containing neurons in the central nervous system. I. Demonstration of monoamines in the cell bodies of brain stem neurons. Acta Physiol Scand Suppl 232: 1–55. 14229500

[15] Jaeger CB, Teitelman G, Joh TH, Albert VR, Park DH, et al (1983) Some neurons of the rat central nervous system contain aromatic-L-amino-acid decarboxylase but not monoamines. Science 219: 1233–1235. 6131537 10.1126/science.6131537

[16] Ugrumov MV (2009) Non-dopaminergic neurons partly expressing dopaminergic phenotype: Distribution in the brain, development and functional significance. J Chem Neuroanat 38: 241–256 10.1016/j.jchemneu.2009.08.004 19698780

[17] Björklund A, Dunnett SB (2007) Dopamine neuron systems in the brain: an update. Trends Neurosci 30: 194–202 10.1016/j.tins.2007.03.006 17408759

[18] Karasawa N, Hayashi M, Yamada K, Nagatsu I, Iwasa M, et al (2007) Tyrosine hydroxylase (TH)- and aromatic-L-amino acid decarboxylase (AADC)-immunoreactive neurons of the common marmoset (Callithrix jacchus) brain: an immunohistochemical analysis. Acta Histochem Cytochem 40: 83–92 10.1267/ahc.06019 17653300 PMC1931487

[19] Björklund A, Dunnett SB (2007) Fifty years of dopamine research. Trends Neurosci 30: 185–187 10.1016/j.tins.2007.03.004 17397938

[20] Ford CP (2014) The role of D2-autoreceptors in regulating dopamine neuron activity and transmission. Neuroscience 10.1016/j.neuroscience.2014.01.025 PMC4108583 24463000

[21] Pappas SS, Behrouz B, Janis KL, Goudreau JL, Lookingland KJ (2008) Lack of D2 receptor mediated regulation of dopamine synthesis in A11 diencephalospinal neurons in male and female mice. Brain Res 1214: 1–10 10.1016/j.brainres.2008.03.010 18462709

[22] Koblinger K, Krajacic A, Nankanishi ST, Whelan PJ (2012) Characterization of A11 neurons projecting to the spinal cord of adult mice. Soc for Neurosci Abstracts: 788.01.

[23] Pappas SS, Tiernan CT, Behrouz B, Jordan CL, Breedlove SM, et al (2010) Neonatal androgen-dependent sex differences in lumbar spinal cord dopamine concentrations and the number of A11 diencephalospinal dopamine neurons. J Comp Neurol 518: 2423–2436 10.1002/cne.22340 20503420 PMC3884812

[24] Paxinos G, Franklin KBJ (2004) The Mouse Brain in Stereotaxic Coordinates. Elsevier Publishing. New York.

[25] Ando-Yamamoto M, Hayashi H, Sugiyama T, Fukui H, Watanabe T, et al (1987) Purification of L-dopa decarboxylase from rat liver and production of polyclonal and monoclonal antibodies against it. J Biochem 101: 405–414. 3584092 10.1093/oxfordjournals.jbchem.a121925

[26] Komori K, Kunimi Y, Yamaoka K, Ito T, Kasahara Y, et al (1992) Semiquantitative analysis of immunoreactivities of tyrosine hydroxylase and aromatic L-amino acid decarboxylase in the locus coeruleus of desipramine-treated mice. Neurosci Lett 147: 197–200. 1362806 10.1016/0304-3940(92)90594-w

[27] Giros B, Jaber M, Jones SR, Wightman RM, Caron MG (1996) Hyperlocomotion and indifference to cocaine and amphetamine in mice lacking the dopamine transporter. Nature 379: 606–612 10.1038/379606a0 8628395

[28] Ciliax BJ, Drash GW, Staley JK, Haber S, Mobley CJ, et al (1999) Immunocytochemical localization of the dopamine transporter in human brain. J Comp Neurol 409: 38–56. 10363710 10.1002/(sici)1096-9861(19990621)409:1<38::aid-cne4>3.0.co;2-1

[29] Lorang D, Amara SG, Simerly RB (1994) Cell-type-specific expression of catecholamine transporters in the rat brain. J Neurosci 14: 4903–4914. 8046459 10.1523/JNEUROSCI.14-08-04903.1994 PMC6577178

[30] Ciliax BJ, Heilman C, Demchyshyn LL, Pristupa ZB, Ince E, et al (1995) The dopamine transporter: immunochemical characterization and localization in brain. J Neurosci 15: 1714–1723. 7534339 10.1523/JNEUROSCI.15-03-01714.1995 PMC6578165

[31] Gainetdinov RR, Wetsel WC, Jones SR, Levin ED, Jaber M, et al (1999) Role of serotonin in the paradoxical calming effect of psychostimulants on hyperactivity. Science 283: 397–401 10.1126/science.283.5400.397 9888856

[32] Skagerberg G, Björklund A, Lindvall O, Schmidt RH (1982) Origin and termination of the diencephalo-spinal dopamine system in the rat. Brain Res Bull 9: 237–244. 7172029 10.1016/0361-9230(82)90136-8

[33] Björklund A, Skagerberg G (1979) Evidence for a major spinal cord projection from the diencephalic A11 dopamine cell group in the rat using transmitter-specific fluorescent retrograde. Brain Res Nov 9;177 1: 170–5. 10.1016/0006-8993(79)90927-2 497819

[34] Ridet I, Privat A (2000) Volume transmission. Trends Neurosci 23: 58–59. 10.1016/s0166-2236(99)01523-4 10652543

[35] Ridet JL, Rajaofetra N, Teilhac JR, Geffard M, Privat A (1993) Evidence for nonsynaptic serotonergic and noradrenergic innervation of the rat dorsal horn and possible involvement of neuron-glia interactions. Neuroscience 52: 143–157. 8381923 10.1016/0306-4522(93)90189-m

[36] Ridet JL, Tamir H, Privat A (1994) Direct immunocytochemical localization of 5-hydroxytryptamine receptors in the adult rat spinal cord: a light and electron microscopic study using an anti-idiotypic antiserum. J Neurosci Res 38: 109–121 10.1002/jnr.490380114 8057387

[37] Fuxe K, Dahlström AB, Jonsson G, Marcellino D, Guescini M, et al (2010) The discovery of central monoamine neurons gave volume transmission to the wired brain. Prog Neurobiol 90: 82–100 10.1016/j.pneurobio.2009.10.012 19853007

[38] Jones SR, Gainetdinov RR, Jaber M, Giros B, Wightman RM, et al (1998) Profound neuronal plasticity in response to inactivation of the dopamine transporter. Proc Natl Acad Sci USA 95: 4029–4034. 9520487 10.1073/pnas.95.7.4029 PMC19957

[39] Morón JA, Brockington A, Wise RA, Rocha BA, Hope BT (2002) Dopamine uptake through the norepinephrine transporter in brain regions with low levels of the dopamine transporter: evidence from knock-out mouse lines. J Neurosci 22: 389–395. 11784783 10.1523/JNEUROSCI.22-02-00389.2002 PMC6758674

[40] Borgkvist A, Malmlöf T, Feltmann K, Lindskog M, Schilström B (2012) Dopamine in the hippocampus is cleared by the norepinephrine transporter. Int J Neuropsychopharmacol 15: 531–540 10.1017/S1461145711000812 21669025

[41] Kawano M, Kawasaki A, Sakata-Haga H, Fukui Y, Kawano H, et al (2006) Particular subpopulations of midbrain and hypothalamic dopamine neurons express vesicular glutamate transporter 2 in the rat brain. J Comp Neurol 498: 581–592 10.1002/cne.21054 16917821

[42] Feenstra MGP, Botterblom MHA, van Uum JFM (2002) Behavioral arousal and increased dopamine efflux after blockade of NMDA-receptors in the prefrontal cortex are dependent on activation of glutamatergic neurotransmission. Neuropharmacology 42: 752–763. 12015201 10.1016/s0028-3908(02)00029-1

[43] Howland JG, Taepavarapruk P, Phillips AG (2002) Glutamate receptor-dependent modulation of dopamine efflux in the nucleus accumbens by basolateral, but not central, nucleus of the amygdala in rats. J Neurosci 22: 1137–1145. 11826142 10.1523/JNEUROSCI.22-03-01137.2002 PMC6758508

[44] Thorpe AJ, Clair A, Hochman S, Clemens S (2011) Possible sites of therapeutic action in restless legs syndrome: focus on dopamine and α2δ ligands. Eur Neurol 66: 18–29 10.1159/000328431 21709418

[45] Clemens S, Belin-Rauscent A, Simmers J, Combes D (2012) Opposing modulatory effects of D1- and D2-like receptor activation on a spinal central pattern generator. J Neurophysiol 107: 2250–2259 10.1152/jn.00366.2011 22262823

[46] Clemens S, Hochman S (2004) Conversion of the modulatory actions of dopamine on spinal reflexes from depression to facilitation in D3 receptor knock-out mice. J Neurosci 24: 11337–11345 10.1523/JNEUROSCI.3698-04.2004 15601940 PMC2731231

[47] Humphreys JM, Whelan PJ (2012) Dopamine exerts activation-dependent modulation of spinal locomotor circuits in the neonatal mouse. J Neurophysiol 108: 3370–3381 10.1152/jn.00482.2012 22993259

[48] Barrière G, Mellen N, Cazalets J-R (2004) Neuromodulation of the locomotor network by dopamine in the isolated spinal cord of newborn rat. Eur J Neurosci 19: 1325–1335 10.1111/j.1460-9568.2004.03210.x 15016090

[49] Whelan P, Bonnot A, O'Donovan MJ (2000) Properties of rhythmic activity generated by the isolated spinal cord of the neonatal mouse. J Neurophysiol 84: 2821–2833. 11110812 10.1152/jn.2000.84.6.2821

[50] Jiang Z, Carlin KP, Brownstone RM (1999) An in vitro functionally mature mouse spinal cord preparation for the study of spinal motor networks. Brain Res 816: 493–499. 9878874 10.1016/s0006-8993(98)01199-8

[51] Kiehn O, Kjaerulff O (1996) Spatiotemporal characteristics of 5-HT and dopamine-induced rhythmic hindlimb activity in the in vitro neonatal rat. J Neurophysiol 75: 1472–1482. 8727391 10.1152/jn.1996.75.4.1472

[52] Gordon IT, Whelan PJ (2006) Monoaminergic control of cauda-equina-evoked locomotion in the neonatal mouse spinal cord. J Neurophysiol 96: 3122–3129 10.1152/jn.00606.2006 16956991

[53] Christie KJ, Whelan PJ (2005) Monoaminergic establishment of rostrocaudal gradients of rhythmicity in the neonatal mouse spinal cord. J Neurophysiol 94: 1554–1564 10.1152/jn.00299.2005 15829596

[54] Madriaga MA, McPhee LC, Chersa T, Christie KJ, Whelan PJ (2004) Modulation of locomotor activity by multiple 5-HT and dopaminergic receptor subtypes in the neonatal mouse spinal cord. J Neurophysiol 92: 1566–1576 10.1152/jn.01181.2003 15163678

[55] Seth P, Gajendiran M, Maitra KK, Ross HG, Ganguly DK (1993) Evidence for D1 dopamine receptor-mediated modulation of the synaptic transmission from motor axon collaterals to Renshaw cells in the rat spinal cord. Neurosci Lett 158: 217–220. 8233099 10.1016/0304-3940(93)90268-p

[56] Han P, Whelan PJ (2009) Modulation of AMPA currents by D(1)-like but not D(2)-like receptors in spinal motoneurons. Neuroscience 158: 1699–1707 10.1016/j.neuroscience.2008.11.040 19110039

[57] Maitra KK, Seth P, Thewissen M, Ross HG, Ganguly DK (1993) Dopaminergic influence on the excitability of antidromically activated Renshaw cells in the lumbar spinal cord of the rat. Acta Physiol Scand 148: 101–107. 8352022 10.1111/j.1748-1716.1993.tb09538.x

[58] Gerin C, Privat A (1998) Direct evidence for the link between monoaminergic descending pathways and motor activity: II. A study with microdialysis probes implanted in the ventral horn of the spinal cord. Brain Res 794: 169–173. 9630613 10.1016/s0006-8993(98)00278-9

[59] Lapointe NP, Rouleau P, Ung R-V, Guertin PA (2009) Specific role of dopamine D1 receptors in spinal network activation and rhythmic movement induction in vertebrates. J Physiol (Lond) 587: 1499–1511 10.1113/jphysiol.2008.166314 19204052 PMC2678221

[60] Sickles AE, Stehouwer DJ, van Hartesveldt C (1992) Dopamine D1 and D2 antagonists block L-dopa-elicited air-stepping in neonatal rats. Brain Res Dev Brain Res 68: 17–22. 1387836 10.1016/0165-3806(92)90243-p

[61] McCrea AE, Stehouwer DJ, van Hartesveldt C (1997) Dopamine D1 and D2 antagonists block L-DOPA-induced air-stepping in decerebrate neonatal rats. Brain Res Dev Brain Res 100: 130–132. 9174256 10.1016/s0165-3806(97)00027-8

[62] Gozal EA, Hayes HB, Hochman S (2008) Effects of the Trace Amines on Hindlimb Motor Output in the Neonatal Rat Spinal Cord. Soc Neurosci Abstracts: 575.11.

